# Digitale Gesundheitskompetenz der Bevölkerung in Deutschland: Aktueller Stand, Konzepte und Herausforderungen

**DOI:** 10.1007/s00103-024-03841-5

**Published:** 2024-02-05

**Authors:** Julia Dratva, Doris Schaeffer, Hajo Zeeb

**Affiliations:** 1 Institut für Public Health, ZHAW Zürcher Hochschule für Angewandte Wissenschaften, Katharina-Sulzer-Platz 9, 8400 Winterthur, Schweiz; 2 Fakultät Medizin, Universität Basel, Basel, Schweiz; 3 Fakultät für Gesundheitswissenschaften, Universität Bielefeld, Bielefeld, Deutschland; 4 Leibniz ScienceCampus Digital Public Health, Leibniz-Institut für Präventionsforschung und Epidemiologie – BIPS, Bremen, Deutschland; 5 Health Sciences Bremen, Universität Bremen, Bremen, Deutschland

**Keywords:** Digitale Gesundheitskompetenz, E‑Health, Digitale Transformation, Messinstrumente, Digital health literacy, eHealth, Digital transformation, Measurement tools

## Abstract

Eine wesentliche Voraussetzung für eine erfolgreiche digitale Transformation des Gesundheitswesens ist eine gut ausgeprägte digitale Gesundheitskompetenz (DGK) der Bevölkerung. DGK ist die Fähigkeit zum Umgang mit gesundheitsbezogenen digitalen Informationen und Informationsmöglichkeiten mit dem Ziel, Gesundheit und Wohlbefinden für sich selbst und sein Umfeld zu fördern und zu erhalten. Der Artikel beleuchtet die Diskussion über DGK, vorhandene Studien und die darin verwendeten Messinstrumente sowie die Datenlage in Deutschland und erörtert aktuelle Herausforderungen.

DGK besteht aus verschiedenen Teilkompetenzen, die aktuelle digitale Informationsverhalten, -möglichkeiten und -risiken widerspiegeln. Die Datenlage ist, aufgrund unterschiedlicher Studiendesigns und -instrumente, sehr heterogen, was die Aussagekraft limitiert. Zwei repräsentative Studien, HLS-GER 2 der Universität Bielefeld sowie die Studie der AOK Rheinland/Hamburg und des Leibniz-WissenschaftsCampus, weisen trotz unterschiedlicher Methoden auf einen hohen Anteil von Menschen mit geringer DGK hin. National wie international zeigt sich, dass die DGK einem sozialen Gradienten unterliegt und mit Bildungsniveau, Sozialstatus, finanzieller Deprivation und Alter assoziiert ist.

Die DGK ist in Deutschland den vorliegenden Daten zufolge noch unzureichend; somit besteht ein großer Handlungsbedarf. Erforderliche gesetzliche Rahmenbedingungen sind gegeben, dennoch fehlt es an verlässlichen finanziellen Ressourcen ebenso an einer soliden Datengrundlage auf Bevölkerungsebene zu DGK. Damit ließen sich Vulnerabilitätsfaktoren identifizieren und die Implementation von Maßnahmen vorbereiten und evaluieren. Zudem bedarf es einer vertiefenden konzeptionellen Diskussion zur DGK, die an das etablierte Gesundheitskompetenzkonzept anknüpft und auch die gesundheitsbezogene Infodemie und ihre Folgen für die DGK aufgreift.

## Abstract (en)

A key prerequisite for the successful digital transformation of the healthcare system is a well-developed level of digital health literacy (DHL) among the population. DHL is the ability to deal with health-relevant digital information and information options with the aim of promoting and maintaining health and well-being for oneself and one’s environment. This article examines the discussions about digital health literacy, the existing studies and measurement tools used in them, the data situation in Germany, and current challenges.

DHL consists of various sub-competencies that reflect current digital information behavior, opportunities, and risks. The data situation is very heterogeneous due to different study designs and instruments, which limits the informative value. Two representative studies, HLS-Ger‑2 by Bielefeld University and the study by AOK Rheinland/Hamburg and the Leibniz-WissenschaftsCampus, both indicate a high proportion of people with low DHL despite different methods. Both nationally and internationally, DHL is subject to a social gradient and is associated with educational level, social status, financial deprivation, and age.

According to the current empirical data, the acquisition of DHL in Germany is still insufficient, so there is a great need for action. The necessary legal framework conditions have been established, but there is still a lack of reliable and financial resources, as well as a solid data basis on DHL at population level. This is essential to identify vulnerability factors and to prepare and evaluate the implementation of measures. In addition, there is a need for an in-depth conceptual discussion on DHL that builds on the established health literacy concept and addresses the emerging health-related infodemic and its consequences for DHL.

## Einleitung

Die digitale Transformation ist auch im deutschen Gesundheitssystem ein wichtiges Thema, was unter anderem daran sichtbar wird, dass in den letzten Jahren eine Vielzahl von Gesetzentwürfen auf den Weg gebracht wurde, um die Digitalisierung im Gesundheitswesen voranzutreiben. Allein in der 19. Legislaturperiode von 2017–2021 wurden 6 entsprechende Gesetze verabschiedet; im August 2023 wurde daran anknüpfend ein Gesetz zur Beschleunigung der Digitalisierung des Gesundheitswesens beschlossen [1]. Dennoch schreitet die Digitalisierung nur sehr langsam voran, wie seit Langem beklagt wird: In einem 2019 publizierten internationalen Vergleich anhand eines mehrdimensionalen Digital-Health-Indikators belegte Deutschland unter 17 analysierten Ländern (14 EU, 3 weitere OECD-Mitgliedsländer) nur Platz 16 [2]. Eine 3 Jahre später publizierte Studie [3] bestätigt dies und zeigt, dass Deutschland bei der Schaffung der notwendigen Grundlagen und der Umsetzung der Digitalisierung weiterhin nur schleppend vorankommt. Innovationen wie Videosprechstunden, elektronische Patient:innenakte, elektronische Rezepte oder digitale Gesundheits-Apps (DIGAs) sind nach wie vor wenig nutzer:innenfreundlich und gebrauchstauglich – nicht zuletzt aufgrund mangelnder partizipativer Einbindung von Versicherten, Patient:innen und Stakeholdern in Deutschland [3, 4]. Darüber hinaus ist die Digitalisierung für Public Health insgesamt von großer Bedeutung, u. a. weil immer mehr Daten, Maßnahmen und Angebote der Prävention und Gesundheitsförderung digital verfügbar sind oder vermittelt werden.

Als eine wesentliche Voraussetzung für eine erfolgreiche digitale Transformation ist die Kompetenz der Bevölkerung und auch der Gesundheitsprofessionen/-berufe anzusehen, mit dem Digitalisierungsschub und der damit verbundenen Veränderung und Verlagerung vielfältiger Informationen und Prozesse in den digitalen Raum erfolgreich umzugehen. Allerdings deutet die bisher vorliegende Evidenz darauf hin, dass es um die digitale Gesundheitskompetenz (DGK) nicht gut bestellt ist. Auch wenn in fast allen Bevölkerungsgruppen mittlerweile eine umfassende Abdeckung mit Internetverbindungen und Smartphones vorliegt und ein enormes Wachstum gesundheitsbezogener Apps zu beobachten ist – dies allein reicht nicht, um Verbesserungen der DGK zu erreichen und gesundheitskompetent agieren zu können. Während der COVID-19-Pandemie ist zudem deutlich geworden, wie wichtig die Kompetenz zum Umgang mit digitalen Gesundheitsinformationen ist, sei es, um eine Infektion zu prävenieren, oder aber, um in Zeiten einer Informationsüberflutung (Infodemie [5]) vertrauenswürdige Informationen zu gewinnen und gesundheitsbezogenen Falschinformationen entgegenzutreten. Die Infodemie ist durch die fortschreitende Digitalisierung erheblich intensiviert und beschleunigt worden, was sich während der COVID-19-Pandemie z. B. in der unüberschaubaren Menge von Informationsangeboten und gesundheitsbezogenen Kommunikationsbeiträgen jeder Art insbesondere in den sozialen Medien zeigte. Die DGK ist zudem unabdingbar, um in angemessener und partizipativer Weise die sich mittlerweile bietenden Möglichkeiten in Digital Public Health besser nutzen zu können. Daher muss es deutlich intensivere Anstrengungen geben, um die DGK zu stärken und auch um die dazu erforderliche Datenbasis zu schaffen. Der vorliegende Artikel beleuchtet zunächst das Konzept der DGK, zeigt dann die Entwicklung bei der empirischen Messung der DGK auf, gibt anschließend anhand von 2 nationalen Studien Einblick in die DGK der Bevölkerung in Deutschland und endet mit einer kritischen Diskussion der Forschung und Förderung der DGK.

### Von E-Health Literacy zu Digital Health Literacy – konzeptionelle Einordnung

Das E‑Health-Literacy-Konzept (eHL), welches zunächst die Diskussion bestimmte, entstand in den späten 1990er-Jahren im Zuge des informationstechnischen Fortschritts und befasste sich mit der „Nutzung elektronischer Technologien im Gesundheitswesen, in der Gesundheitsversorgung und im öffentlichen Gesundheitswesen“ [6]. Mit der raschen Ausweitung digitaler Informationsmöglichkeiten vollzog sich ein Begriffs- und Konzeptwechsel, im Zuge dessen der Begriff eHL zunehmend durch Digital Health Literacy bzw. digitale Gesundheitskompetenz (DGK) abgelöst wurde [7]. DGK unterscheidet sich von eHL und meint die Fähigkeit, mit gesundheitsbezogenen Informationen aus unterschiedlichsten digitalen Informationsquellen umgehen zu können – nicht allein dem Internet, sondern auch anderen digitalen gesundheitsbezogenen Informationsmöglichkeiten von Mobile Health (M-Health) bis hin zur künstlichen Intelligenz [8, 9].

**Figure 1:**
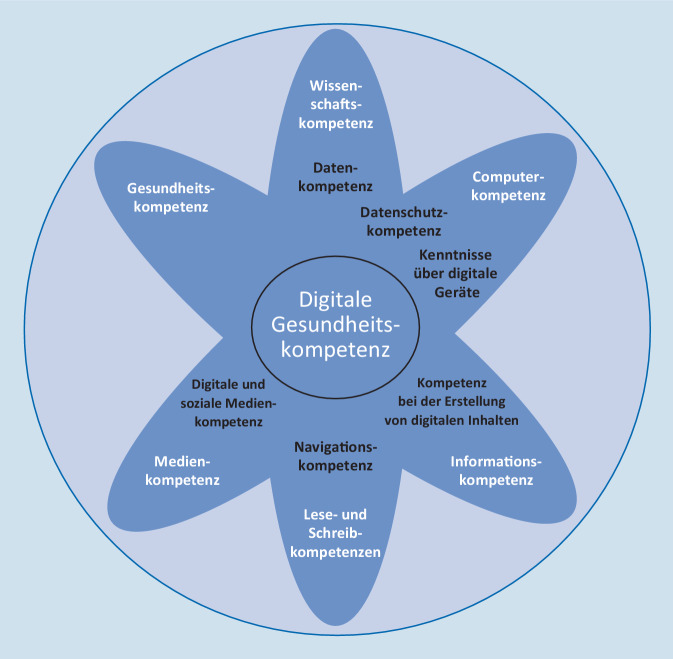

Ebenso wie eHL orientierte sich das DGK-Konzept weitgehend am Verständnis der generischen/allgemeinen Gesundheitskompetenz (GK; [10, 11]). Das Lily-Model von Norman und Skinner beschrieb erstmals eine Reihe von Teilkompetenzen, die für eine digitale Kompetenz als essentiell erachtet wurden (Abb. 1), so etwa Kompetenzen betreffend Computernutzung (Computer Literacy), kritischen Umgang mit Informationen, Medien und Medieninhalten (Information Literacy, Media Literacy), Wissen und Verständnis wissenschaftlicher Grundlagen, Konzepte und Prozesse (Science Literacy) neben traditionellen Lese- und Schreib-Kompetenzen (Traditional Literacy) und Gesundheitskompetenz (Health Literacy; [11]). Neuere DGK-Konzepte inkludieren auch die prosumierende Rolle der Bevölkerung, die mit der Entwicklung des World Wide Web und der sozialen Medien möglich wurde. Denn längst wird nicht mehr nur digitale Information konsumiert, sondern auch selbst produziert und bereitgestellt. Zudem tauschen sich Bürger:innen digital aus und informieren sich untereinander. Eine aktuelle Definition von Griebel et al. [12] schließt diese neuen Kompetenzen und die Verwendung digitaler Technologien im Kontext Gesundheit ein und weist zudem darauf hin, dass die individuellen Komponenten der DGK immer auch in Relation zu kontextuellen und sozialen Faktoren zu verstehen sind (Infobox 1).

Zunehmend wird auch die Frage diskutiert, ob DGK als Spezifikum definiert und konzeptualisiert werden sollte oder als Teil der allgemeinen GK und des entsprechenden Konzepts anzusehen ist. Für Letzteres spricht, dass gesundheitsbezogene Informationen mittlerweile überwiegend digital zur Verfügung stehen und die Bedeutung digitaler Informationsquellen rasant gewachsen ist. Schon heute stehen sie an erster oder zweiter Stelle der genutzten Informationsquellen [13, 14]. Besonderheiten der DGK wie die erforderlichen computer- bzw. internetbezogenen Fähigkeiten, Datenschutzkompetenz oder die Fähigkeit, mit Austauschplattformen und mobilen Gesundheits-Applikationen (M-Health) umzugehen, lassen sich gut mit dem GK-Konzept in Einklang bringen. Auch um eine konzeptionelle Spaltung zu vermeiden, wird dafür plädiert, GK und DGK eng verknüpft zu betrachten, was auch der empirischen Befundlage entspricht. Dabei liegt es nahe, auch in Deutschland an das GK-Verständnis von Sørensen et al. [15] anzuknüpfen, das sich in Europa weitgehend durchgesetzt hat. Danach ist GK als Fähigkeit zu verstehen, gesundheitsbezogene Informationen in unterschiedlicher Form – und somit auch in digitaler Form – finden, verstehen, einschätzen und nutzen zu können, um tragfähige Entscheidungen für das eigene Gesundheitshandeln zu treffen. Die Definition nach Sørensen entspricht weitgehend der neueren Definition von Nutbeam und Muscat (siehe Infobox 1), wenngleich Letztere organisationale und systemische Aspekte deutlicher formulieren. Zusammengefasst lässt sich DGK somit als Fähigkeit zum Umgang mit gesundheitsbezogenen digitalen Informationen und Informationsmöglichkeiten definieren, die von strukturellen Bedingungen und der Verfügbarkeit von (technischen und anderen) Ressourcen mitgestaltet wird.

### Messung digitaler Gesundheitskompetenz

Das erste wissenschaftlich validierte Instrument zur Messung der digitalen Gesundheitskompetenz, eHealth Literacy Scale (eHEALS), stammt von Norman und Skinner [7]. Es wurde im Lauf der Zeit erweitert, weil die technologische Entwicklung auf weitere Teilkompetenzen verwies. Gleichwohl haben die Grundkompetenzen weiterhin Bestand. Hier deutet sich eine Entwicklung an, die aktuell insgesamt in der Gesundheitskompetenzforschung zu beobachten ist. Es werden immer neue Instrumente für einzelne GK-Bereiche entwickelt, die faktisch als Teilkompetenzen eines umfassenden GK-Konzeptes zu verstehen sind. Oft fehlt ihnen jedoch die Rückbindung an das GK-Konzept und damit meist auch die Anschlussfähigkeit an den dortigen Konzept- und Methodendiskurs.

Dies zeigt sich auch mit Blick auf die vielen existierenden DGK-Messinstrumente. So liegen unterschiedliche Instrumente zur Ermittlung der generellen DGK vor ebenso für einzelne Patient:innengruppen, unterschiedliche Altersgruppen oder Kontexte. Aufgrund der unterschiedlichen Zielsetzungen, aber auch wegen des technologischen Fortschritts, unterscheiden sie sich zudem in den gemessenen Dimensionen [17, 18]. Neuere Instrumente, wie das **„**Digital Health Literacy Instrument**“** (DHLI; [19]), adressieren die sogenannten Health‑2.0‑Kompetenzen sowie Kompetenzen der digitalen Kommunikation, des Erstellens eigener Inhalte, des Schutzes der eigenen Privatsphäre sowie leistungsbezogene Elemente (Perfomance-based Items). Allerdings besteht nicht nur die Notwendigkeit, DGK-Instrumente an neue technologische Anwendungen anzupassen, sondern auch an die gestiegenen operativen digitalen Kompetenzen, wodurch manche Items mit der Zeit nicht mehr sinnvoll sind. Die große Zahl an unterschiedlichen konzeptionellen Zugängen und Instrumenten ist dennoch durchaus kritisch zu sehen, da sie einer einheitlichen und aussagekräftigen Datenbasis zur DGK der Bevölkerung, wie sie für eine tragfähige und gezielte Stärkung der DGK nötig ist, entgegensteht.

### Digitale Gesundheitskompetenz der Bevölkerung in Deutschland – 2 Studienbeispiele

Im deutschsprachigem Raum sind erst in den letzten Jahren Untersuchungen zur DGK erschienen [20–24]. Nachfolgend werden exemplarisch 2 nationale Studien zur DGK vorgestellt. Beide sind große populationsbasierte Studien mit Fokus auf der erwachsenen Bevölkerung, nutzen aber unterschiedliche methodische Vorgehensweisen und Instrumente und sind somit nur eingeschränkt vergleichbar.

Die unbefriedigende Forschungslage gab den Anstoß, das Thema DGK 2018 auch in den zweiten „Health Literacy Survey Germany“ (HLS-GER 2) aufzunehmen, der ein Teil des „Health Literacy Population Survey“ (HLS19) des „WHO Action Network on Measuring Population and Organizational Health Literacy“ (M-POHL) mit 17 Ländern ist [9, 25]. Dort wurde DGK als Teil der allgemeinen GK [15] definiert und ein damit in Übereinstimmung stehendes Messinstrument entwickelt, das auf dem DHLI [19] beruht und aus forschungspragmatischen Gründen, u. a. einer zumutbaren Länge des Fragebogens, gekürzt wurde. Das Messinstrument besteht aus insgesamt 10 Items, von denen 8 die selbst eingeschätzten Schwierigkeiten beim Finden, Verstehen, Beurteilen und Anwenden von digitalen Gesundheitsinformationen erfragen. 2 weitere Items beziehen sich auf die digitale Kommunikation mit Gesundheitspersonal und die damit verbundenen subjektiven Herausforderungen (ausführlicher, auch zur Auswertungsstrategie [8, 24, 26]). In Deutschland wurde in dem vom Bundesministerium für Gesundheit geförderten HLS-GER 2 eine bundesweit repräsentative Stichprobe von *n* = 2151 Personen vor der COVID-19-Pandemie und eine kleinere Stichprobe (*n* = 540) im Rahmen einer Zusatzerhebung während der Pandemie persönlich befragt [25, 27].

In Deutschland verfügen dem HLS-GER 2 zufolge 76 % über eine geringe DGK (9 % problematische und 66 % inadäquate DGK; [25]). Anders formuliert: 3 Viertel der Bevölkerung in Deutschland haben große Schwierigkeiten, mit digitalen Informationen umzugehen. Mit Abstand am schwersten fällt es den Befragten, die Vertrauenswürdigkeit und Neutralität digitaler Gesundheitsinformationen einzuschätzen. Dies ist kein Spezifikum in Deutschland. Zum gleichen Ergebnis kommen auch die anderen 13 Länder, die die DGK im HLS19 erhoben haben [9]. Dies dürfte als Hinweis auf die sich weltweit vollziehende Infodemie mit ihrem undurchschaubaren Nebeneinander von seriösen und unseriösen Informationen zu verstehen sein, welche überaus herausfordernd für den Umgang mit digitalen Informationen ist. International wie national zeigt sich zudem, dass die DGK – wie auch die allgemeine GK – einem sozialen Gradienten unterliegt und stark durch Bildungsniveau, Sozialstatus, finanzielle Deprivation und Alter beeinflusst wird [9, 25, 28]. Anders formuliert: Menschen aus niedrigen Bildungs- und Sozialschichten und im höheren Lebensalter weisen in Deutschland eine besonders geringe DGK auf und werden deshalb als vulnerabel bezeichnet. Menschen mit Migrationserfahrung, die lange Zeit als vulnerabel für geringe GK und – so war anzunehmen – auch geringe DGK galten, sind es dem HLS-GER 2 [25] und auch dem spezifisch auf Menschen mit ex-sowjetischem und türkischem Migrationshintergrund ausgerichteten HLS-MIG [28] zufolge nicht, sogar gegenteilig: Sie weisen eine bessere GK und DGK als die Allgemeinbevölkerung auf und nutzen digitale Informationsmöglichkeiten intensiver.

Generell ist die Nutzungshäufigkeit digitaler Informationsmöglichkeiten in Deutschland nicht sehr hoch: Internetseiten werden nach dem HLS-GER 2 am häufigsten genutzt (von insgesamt 64 % der Befragten), soziale Medien von 38 %, digitale Geräte von 31,5 % und Gesundheits-Apps nur von 21 %. Noch geringer sind die Nutzungszahlen bei digitalen Interaktions- und Kommunikationsmöglichkeiten, damit ist die Verwendung von E‑Mail-Kommunikation oder auch von Chats und Blogs auf gesundheitsbezogenen Webseiten gemeint. Lediglich 16 % der Befragten geben an, davon Gebrauch zu machen [25, 28]. In Übereinstimmung mit den Ergebnissen zur DGK nehmen vulnerable Gruppen mit geringerer GK vergleichsweise seltener digitale Informationsmöglichkeiten in Anspruch [25, 28]. Dem relationalen Modell von GK zufolge [29] weist dies deutlich auf die sie umgebenden Kontexte und Strukturbedingungen und möglicherweise auf eingeschränktere Optionsspielräume hin. Zugleich deutet es auf die aktuell existierende digitale Spaltung hin, die weiterhin eine zentrale Rolle bei der DGK und auch im Bereich Digital Public Health spielt (siehe auch Beitrag von Brand et al. in diesem Themenheft).

Eine weitere, bundesweit repräsentative Untersuchung zur DGK wurde vom AOK Bundesverband, der AOK Rheinland/Hamburg und dem Leibniz-WissenschaftsCampus kurz nach dem Beginn der COVID-19-Pandemie im Herbst 2020 durchgeführt [30]. Online befragt wurden 8500 Teilnehmende eines etablierten Panels, dabei wurde der DHLI-Fragebogen [19] mit 21 Items zu 7 Dimensionen (Teil-Literacies) der DK in (übersetzter) Originalform genutzt. Anders als im HLS-GER 2 wurden daher auch technisch-operative Fähigkeiten z. B. im Umgang mit Computern und bei der Erstellung eigener Inhalte eingeschlossen, sodass auf Inhaltsebene keine Vergleichbarkeit mit HLS-GER 2 vorliegt. Das gilt auch in methodischer Hinsicht: Für die Auswertung wurde die DGK ähnlich wie in HLS-GER 2 in 4 Kategorien (gering, moderat, hoch, sehr hoch) eingeteilt, wobei die optimalen Cut-off-Punkte der 4‑Punkte-Skala mittels eines Regressionsansatzes aus den Daten ermittelt wurden. Im Ergebnis wiesen 52 % der Befragten eine geringe oder moderate DGK auf, der Anteil derjenigen mit geringer (also der Kategorie „inadäquat“ des HLS-GER 2 entsprechend) war mit 28 % jedoch deutlich niedriger. Dieser Unterschied ist zumindest anteilig durch die vergleichsweise hohen Werte bei den operativen Fähigkeiten erklärbar ebenso durch Unterschiede im methodischen Vorgehen. Zudem kann die repräsentative Panel-Population generell höhere digitale Kompetenzen aufweisen als eine zufällige Bevölkerungsstichprobe. Die Einschätzung digitaler Informationen wies dagegen wie im HLS-GER 2 die niedrigsten Werte auf.

Es liegen Hinweise dafür vor, dass sich das digitale Informationsverhalten mit der Pandemie verändert hat. Seither werden digitale Angebote häufiger genutzt und auch die Nutzerkreise sind größer geworden: Sie haben um rund 8 % zugenommen, wie eine kurz nach Beginn der COVID-19-Pandemie durchgeführte Zusatzerhebung im Rahmen der HLS-GER 2 zeigt [25]. Verbessert hat sich auch die DGK in der ersten Zeit der Pandemie: Der Anteil geringer DGK hat sich um ca. 5 % verringert, bei der inadäquaten DGK sind es sogar fast 9 % und noch mehr bei Menschen mit geringer Bildung [25]. Dies kann als Hinweis darauf angesehen werden, dass eine Verbesserung der DGK auch in recht kurzer Zeit gelingen kann. Jedoch bleibt abzuwarten und zu prüfen, ob diese Veränderungen nachhaltig sind.

### Diskussion und Schlussfolgerung

Digitale Gesundheitskompetenz ist eine zentrale Voraussetzung, um eine erfolgreiche digitale Transformation der Gesundheitssysteme zu gewährleisten und das Potenzial der Digitalisierung auszuschöpfen. Die vorgestellten Daten machen deutlich, dass der Erwerb von DGK nach wie vor schleppend verläuft und großer Handlungsbedarf besteht. International zeigt die HLS-19-Erhebung, dass Deutschland unter den Ländern, die die DGK gemessen haben, den höchsten Anteil an geringer DGK aufweist [9]. Dieser Befund passt in das Gesamtbild des Stands der digitalen Transformation im Gesundheitswesen, der eingangs kurz dargestellt wurde.

Der Vergleich der Daten vor und während der ersten Monate der COVID-19-Pandemie deutet an, dass es in relativ kurzer Zeit möglich war, die DGK zu verbessern – dies besonders bei Menschen mit geringer GK und bei Menschen mit niedriger Bildung, die zu den vulnerablen Gruppen mit hohem Anteil an geringer GK gehören. Verständlich wird dies, wenn man sich vergegenwärtigt, dass in dieser Zeit quasi notgedrungen Gesundheitsinformationen gesucht und rezipiert werden mussten, allein um auf Infektionsgefahren zu reagieren und sich und andere schützen zu können. Dazu stand nach kurzer Zeit ein solches Ausmaß an digitalen Gesundheitsinformationen und Kommunikationsformen zur Verfügung wie nie zuvor. Ob der Trend zu einer erhöhten Nutzung und besseren DGK anhält, ist jedoch ungewiss. Denn die COVID-19-Pandemie stellte eine Ausnahmesituation dar. Die gesellschaftliche Notlage dürfte große Handlungs- und Innovationsbereitschaft erzeugt und der digitalen Transformation einen großen Schub verliehen haben. Mittlerweile wird das Risikopotenzial deutlich geringer eingeschätzt und es besteht die Gefahr, wieder auf „altbewährte“ Verhaltensweisen bei der gesundheitsbezogenen Information und Kommunikation zurückzugreifen. Daher sind die Förderung der DGK und Schaffung digital kompetenter Strukturen weiterhin eine wichtige gesellschaftspolitische Aufgabe, die auch ohne Public-Health-Notlage vorangetrieben werden muss.

Mit dem § 20k SGB V (Sozialgesetzbuch Fünftes Buch), mit dem die Förderung von DGK gesetzlich verankert wurde, ist dazu ein wichtiger Schritt erfolgt, dem sich weitere anschließen müssen. Dies erfordert ein intensiveres Agenda-Setting auf der politischen Ebene ebenso wie bei den umsetzenden Akteur:innen und wichtigen Stakeholdergruppen.

Nicht weniger wichtig ist eine Intensivierung der konzeptionellen und methodischen Diskussion. Faktisch existiert bis heute eine sehr disparate Forschungs- und Datenlage, in der weder ein wirklicher Konsens über den Untersuchungsgegenstand noch über konzeptionelle Grundlagen und geeignete Messinstrumente besteht. Dies führt dazu, dass die aktuelle Datenlandschaft hinsichtlich der Studiendesigns und -instrumente sowie der untersuchten Bevölkerungen nach wie vor sehr heterogen ist und es an vergleichbaren Daten mangelt. Dies haben auch die beiden vorgestellten Studien exemplarisch gezeigt. Damit fehlen in Deutschland jedoch nach wie vor wichtige Bedingungen für eine gut begründbare datenbasierte, präferenzsensible Interventionsentwicklung.

Die mangelnde Vergleichbarkeit limitiert zudem den Impact von Studien. Selbst wenn, wie in den vorgestellten Studien, die zentralen Aussagen zwar nahe beieinander liegen, aber nicht identisch sind, bleiben die (erklärbaren) Unterschiede für Politik und Bevölkerung oft schwer verständlich und vermittelbar. Auch ist der Erkenntnisgewinn aus den Daten limitiert. Ein Zusammenführen (Poolen) der Daten mit dem Ziel größerer und aussagekräftigerer Datensätze würde detailliertere Analysen z. B. zu weniger häufigen Einflussfaktoren ermöglichen. Als Evidenzbasis für die Förderung der DGK sind solche großen und populationsbasierten Datensätze essenziell. Denn sie erlauben einerseits Trends zu erfassen und spezifische Populationsgruppen und Einflussfaktoren zu adressieren und andererseits datenbasierte Interventionen und Förderkonzepte zu entwickeln. Sinnvoll ist daher, künftig auch international die Verwendung desselben Instruments bzw. zentraler Kernelemente anzustreben. Dazu hat das WHO Action Network on Measuring Population and Organizational Health Literacy (M-POHL) bereits wichtige Entwicklungen angestoßen.

Gleichzeitig ist es wichtig, Offenheit für die sich verändernden Anforderungen an die DGK und das Voranschreiten der digitalen technologischen Entwicklung – wie etwa im Bereich der künstlichen Intelligenz – zu bewahren und vorliegende Messinstrumente entsprechend zu adaptieren. Nicht weniger wichtig ist es, geeignete Interventionsansätze zur GK und DGK zu entwickeln und umzusetzen und die dazu nötige Implementationsforschung zu fördern.

Zugleich ist erforderlich, auch die Auseinandersetzung über konzeptionelle Fragen zu intensivieren. So stellt sich beispielsweise die Frage, ob es sinnvoll ist, die Ausdifferenzierung von Teilkompetenzen isoliert von der Rückbindung an das GK-Konzept fortzusetzen. Die damit einhergehenden Gefahren dürften eher zur Schwächung als zur Stärkung des Konzepts führen. Zudem belegen die vorliegenden Befunde, wie eng beide Konzepte miteinander verknüpft sind [28].

Während der COVID-19-Pandemie ist deutlich geworden, wie schwierig es ist, mit der Flut an digitalen Informationen und der für sie typischen Mixtur an seriösen und an Falsch- und Fehlinformationen zurechtzukommen. Die Beurteilung der Zuverlässigkeit digitaler Informationen stellt dabei eine der größten Herausforderungen für die Bevölkerung dar. Erneut lässt sich daraus ableiten, wie wichtig die Förderung der DGK ist. Zugleich stellt sich die Aufgabe, die Qualität und Vertrauenswürdigkeit von Gesundheitsinformationen zu verbessern und die systemische, organisationale und professionelle DGK zu fördern. Den Gesundheitseinrichtungen und den darin tätigen Gesundheitsprofessionen kommt eine hohe Bedeutung der Förderung der DGK der Bevölkerung zu. Wollen sie diese anspruchsvolle Aufgabe kompetent wahrnehmen, setzt dies allerdings u. a. eine hohe digitale Kompetenz seitens der Gesundheitsprofessionen voraus. Auch die professionelle GK und DGK zu fördern, stellt eine weitere wichtige, in ihrer Bedeutung bislang unterschätzte Aufgabe dar [31]. Dass dabei innovative, kokreative Ansätze gefordert sind, sei ausdrücklich betont.

Abschließend sei erneut darauf hingewiesen, dass auch die DGK sozial ungleich verteilt ist [32–34]. Bildung, Sozialstatus und Alter sind wiederkehrend mit niedriger Ausprägung der digitalen Gesundheitskompetenz assoziiert. Ebenfalls wiederkehrend zeigen Studien, dass diese Merkmale mit schlechterer Gesundheit und ungesünderem Verhalten verbunden sind und diese Gruppen größeren Gesundheitsrisiken ausgesetzt sind. Qualitativ hochwertige und relevante Forschung zu DGK muss daher anstreben, sozioökonomische Einflussfaktoren detailliert einzubeziehen und den Blick explizit auf vulnerable Gruppen zu richten. Dass dafür eine dezidierte und verlässliche Forschungsförderung notwendig ist, steht außer Frage.

## Fazit

Eine hohe digitale Gesundheitskompetenz der Bevölkerung scheint ein zentraler Faktor zu sein, um das Potenzial der digitalen Transformation des Gesundheitssystems und der Gesundheitsinformationen zu maximieren. In Deutschland ist die digitale Gesundheitskompetenz der Bevölkerung trotz Hinweisen auf positive Entwicklungen während der COVID-19-Pandemie unzureichend. Bei der Förderung der digitalen Gesundheitskompetenz sowie bei der Schaffung von digitalen Informations- und Interaktionsangeboten sollten die Maßnahmen und deren Implementierung wissenschaftlich begründet und begleitet werden. Im Kontext der Stärkung der DGK ist die Sicherstellung einer digitalen Chancengleichheit von größter Wichtigkeit, um nicht neue soziale Gradienten oder Risikofaktoren zu etablieren.

### Infobox 1 Aktuelle Definition der generellen und der digitalen Gesundheitskompetenz

**Health Literacy** (Health promotion glossary, Nutbeam und Muscat 2021 [16])

„Health literacy represents the personal knowledge and competencies which accumulate through daily activities, social interactions and across generation. Personal knowledge and competencies are mediated by the organizational structures and availability of resources which enable people to access, understand, appraise, and use information and services in ways which promote and maintain good health and wellbeing for themselves and those around them.“

Gesundheitskompetenz steht für das persönliche Wissen und die Kompetenzen, die sich durch tägliche Aktivitäten, soziale Interaktionen und über Generationen hinweg ansammeln. Persönliche Kenntnisse und Kompetenzen werden durch die organisatorischen Strukturen und die Verfügbarkeit von Ressourcen vermittelt, die es den Menschen ermöglichen, Informationen und Dienstleistungen so zu nutzen, zu verstehen, zu bewerten und zu verwenden, dass sie Gesundheit und Wohlbefinden für sich selbst und ihr Umfeld fördern und erhalten.

**Digital Health Literacy **(Griebel et al. 2018 [12])

„… a dynamic and context specific set of individual and social factors, as well as consideration of technological constraints in the use of digital technologies to search, acquire, comprehend, appraise, communicate, apply and create health information in all contexts of health care with the goal of maintaining of improving the quality of life throughout the lifespan“.

… eine dynamische und kontextspezifische Anzahl individueller und sozialer Faktoren sowie die Berücksichtigung technologischer Bedingungen bei der Nutzung digitaler Technologien für die Suche, den Erwerb, das Verstehen, die Bewertung, die Kommunikation, die Anwendung und die Kreation von Gesundheitsinformationen in allen Bereichen der Gesundheitsversorgung mit dem Ziel, die Lebensqualität über die gesamte Lebensspanne hinweg zu erhalten oder zu verbessern.

(*Übersetzungen aus dem Original durch die Autoren*)
